# Dating the emergence of dairying by the first farmers of Central Europe using ^14^C analysis of fatty acids preserved in pottery vessels

**DOI:** 10.1073/pnas.2109325118

**Published:** 2022-10-17

**Authors:** Emmanuelle Casanova, Timothy D. J. Knowles, Alex Bayliss, Mélanie Roffet-Salque, Volker Heyd, Joanna Pyzel, Erich Claßen, László Domboróczki, Michael Ilett, Philippe Lefranc, Christian Jeunesse, Arkadiusz Marciniak, Ivo van Wijk, Richard P. Evershed

**Affiliations:** ^a^Organic Geochemistry Unit, School of Chemistry, Bristol BS8 1TS, United Kingdom;; ^b^Bristol Radiocarbon Accelerator Mass Spectrometry Facility, University of Bristol, Bristol BS8 1UU, United Kingdom;; ^c^Scientific Dating, Historic England, Cannon Bridge House, London EC4R 2YA, United Kingdom;; ^d^Department of Cultures/Archaeology, University of Helsinki 00014 Helsinki, Finland;; ^e^Institute of Archaeology and Ethnology, University of Gdańsk PL 80-851 Gdańsk, Poland;; ^f^LVR-State Service for Archaeological Heritage 53115 Bonn, Germany;; ^g^Dobó István Castle Museum, Eger H-3300, Hungary;; ^h^UMR 8215 Trajectoires, Université Paris 1 Panthéon-Sorbonne, 75004 Paris, France;; ^i^UMR 7044/Institut National des Recherches Archéologiques Préventives, University of Strasbourg, 67100 Strasbourg, France;; ^j^UMR 7044, Misha, University of Strasbourg, 67083 Strasbourg, France;; ^k^Institute of Archaeology, Adam Mickiewicz University 61-614 Poznan, Poland;; ^l^Archaeological Research Leiden, Leiden University, Leiden 2333 CC, The Netherlands

**Keywords:** radiocarbon dating, ceramics, dairy residues, Neolithic, Central Europe

## Abstract

Direct, accurate, and precise dating of archaeological pottery vessels is now achievable using a recently developed approach based on the radiocarbon dating of purified molecular components of food residues preserved in the walls of pottery vessels. The method targets fatty acids from animal fat residues, making it uniquely suited for directly dating the inception of new food commodities in prehistoric populations. Here, we report a large-scale application of the method by directly dating the introduction of dairying into Central Europe by the *Linearbandkeramik* (LBK) cultural group based on dairy fat residues. The radiocarbon dates (*n* = 27) from the 54th century BC from the western and eastern expansion of the LBK suggest dairy exploitation arrived with the first settlers in the respective regions and were not gradually adopted later. This is particularly significant, as contemporaneous LBK sites showed an uneven distribution of dairy exploitation. Significantly, our findings demonstrate the power of directly dating the introduction of new food commodities, hence removing taphonomic uncertainties when assessing this indirectly based on associated cultural materials or other remains.

## Significance

Calendrical dating for the introduction of new food commodities affords enhanced understanding of major changes in human food procurement. Here, direct dating of milk residues from the Early Neolithic in Central Europe demonstrates the use of this unique secondary product from animals arrived with the earliest *Linearbandkeramik* settlers in the western (France, the Netherlands, and northwestern Germany) and eastern (Poland) extensions of the cultural group. At a time when most adult humans lacked the lactase-persistence gene variant, the adoption and intensification of a dairy-based economy would have had significant impact on human diet, evolution, and environment.

The introduction of new food commodities into the human diet at the very beginnings of plant and animal domestication is one of the most critical questions in the Neolithization process, having far reaching consequences for human evolution and environmental change. Of major importance is milk exploitation, as it relates to animal domestication but also the ability of adult humans to digest lactose (1, 2). Clearly, identifying the beginnings of the exploitation of domesticated animals for their secondary products (i.e., those obtained during the life of animals, such as milk, wool, or blood) as opposed to primary products (i.e., those obtained by the death of the animal such as meat, skin, teeth, or horn) makes it extremely important to establish when and how dairying began (3, 4). Directly dating the introduction of a new food commodity is nonetheless challenging.

Evidence for dairy exploitation in prehistory can be interpreted from iconography, diagnostic ceramics, or domesticated animal slaughter patterns based on sex and ages (3, 4). Additionally, direct evidence for dairy exploitation can be derived from lipid analyses of food residues preserved in pottery vessels. By determining the stable carbon isotope values of the two fatty acids (FAs) (C_16:0_ and C_18:0_) characteristic of degraded animal fats, dairy products can be distinguished from carcass products (5). Recent combined lipid residue analyses of pottery vessels and animal management assessments based on faunal remains (stable isotopes, butchery practices, kill-off patterns, and calving patterns) have provided invaluable knowledge of early dairying practices at archaeological sites. Currently, the earliest evidence for milk use from lipid residues and faunal assemblages recovered during the Neolithic was found in Anatolia during the 7th millennium BC (6), from several regions in the Balkans, eastern Europe, and the Mediterranean during the 6th millennium BC (7–12), in Saharan Africa (Libya and Algeria) during the 5th millennium BC (13–15), from the beginning of the Neolithic in Britain, Ireland, and Scandinavia during the 4th millennium BC (5, 16–19), and in the Baltic countries during the 3rd millennium BC (16). The dates of the introduction of dairying in these regions have been established largely indirectly based on associated materials (e.g., animal bone collagen, charcoal, charred seeds, etc.) recovered from the same archaeological contexts as the pottery yielding milk fat residues. However, uncertainties exist with indirect dating due to possible intrusion or residuality of datable materials, resulting from the disturbance of archaeological layers and the requirement for the datable materials to be short-lived and truly contemporaneous in date with the pottery vessels containing the dairy residues.

Thus, the application of recently developed methods for the direct dating of lipids from pottery food residues offers a unique approach to obtain accurate and precise dates for the introduction of new food commodities. The direct ^14^C dating of dairy fat residues avoids all the aforementioned uncertainties, offering an unprecedented opportunity to accurately date the start of dairying practices. At the University of Bristol, United Kingdom, we recently reported a method for radiocarbon dating pottery vessels from their absorbed food residues. Our compound-specific radiocarbon analysis (CSRA) approach is based on the isolation of the C_16:0_ and C_18:0_ FAs from the clay matrix and freeing them from exogenous organic contaminants (20, 21). We have successfully applied this approach to a small number of dairy residues from the Libyan Sahara and Central Europe, with one of the oldest dated dairy residues coming from the 6th millennium in the Balkans (11, 22). Hence, this dating method offers the opportunity to directly date residues identified as dairy fats based on the compound-specific δ^13^C values of the C_16:0_ and C_18:0_ FAs, avoiding taphonomic uncertainties arising from dating-associated materials.

In this paper, we focus on the *Linearbandkeramik* (LBK) culture, the first farming society in Central Europe, which emerged and expanded over much of northern Europe in the middle of the 6th millennium BC (23). This culture has been divided into five main phases: Earliest (I), Early (II), Middle (III), Late (IV), and Final (V) LBK, known as the Meier-Arendt chronology, whose timing and evolution differed in the different regions of the LBK (24). Hence, the ceramic phases discussed in the remainder of this paper use the regional and site classifications for the chronology of earliest, early, middle, and Late LBK, which are not necessarily contemporaneous. For example, phase I in Poland and phase I in Cuiry-lès-Chaudardes refer to the Earliest and Late LBK phases, respectively, in the Meier-Arendt chronology.

Dairy residues were identified in varying quantities at LBK sites across Central Europe. Some sites show only a weak dairy signal (1 to 2 potsherds only), while others display much higher recovery, with over 20% of the residues displaying dairy fat molecular and carbon isotope characteristics. These results emphasize the spatial disparity in the exploitation of cattle and caprines for their milk in this period. We do not exclude the possibility that the use of organic containers other than clay vessels for dairy products at some sites may affect the overall dairy lipid recovery observed. Diachronic studies in certain regions also revealed dairy practices evolving from being nonexistent or at very low levels at LBK sites but becoming much more abundant in the following Middle Neolithic cultures [e.g., the Rössen culture in Lower Alsace, France (22) or Funnel Beaker culture at the site of Kopydłowo, Poland (25)]. Dating of dairy residues recovered from the earliest phases of the sites would provide calendar ages for the emergence of dairying between LBK regions based directly on the commodity itself rather than on associated materials. Critically, some sites cannot be dated by conventional materials due to their poor preservation, while at other sites where dairy evidence is scarce, the possibility exists for false-positive signal arising due to stratigraphic perturbations. In reporting here the application of our recently developed CSRA method to a wide range of potsherds, we begin to resolve the timing of appearance of dairying practices by LBK farmers during the Neolithic in the diverse regions of the settlements.

## Results

### Pottery Selection.

A total of 4,327 pottery vessels from 70 LBK settlements have been previously analyzed for their lipid residues. From those, 1,413 pottery vessels (33% of potsherds analyzed) from 61 sites (87% of the sites) were shown to contain animal fat residues, of which 353 vessels (25% of potsherds with animal fats or 8% of the total) from 46 sites (66% of the sites) were identified as containing dairy fats [Δ^13^C ≤ −3.1 ‰ (5, 7, 14)]. For ^14^C dating, we focused on sites where dairy signals were detected in potsherds, even if this was limited to one to two vessels. Wherever possible, pottery was selected according to the following criteria: 1) potsherds with dairy residues from the oldest LBK phase of the settlement (identified by pottery typology and stratigraphy); 2) potsherds with lipid concentrations >250 µg ⋅ g^−1^ but ideally >500 µg ⋅ g^−1^ to allow sufficient C for a radiocarbon date from a minimum amount of clay from a single pot (21); 3) potsherds of 1 to 20 g to provide sufficient clay fabric; and 4) a minimum of two potsherds per context or phase; if only one dairy residue was available for ^14^C dating, the sampling was complemented by a ruminant/nonruminant carcass fat from the same context/phase to provide dates on contemporaneous vessels.

**Fig. 1.**
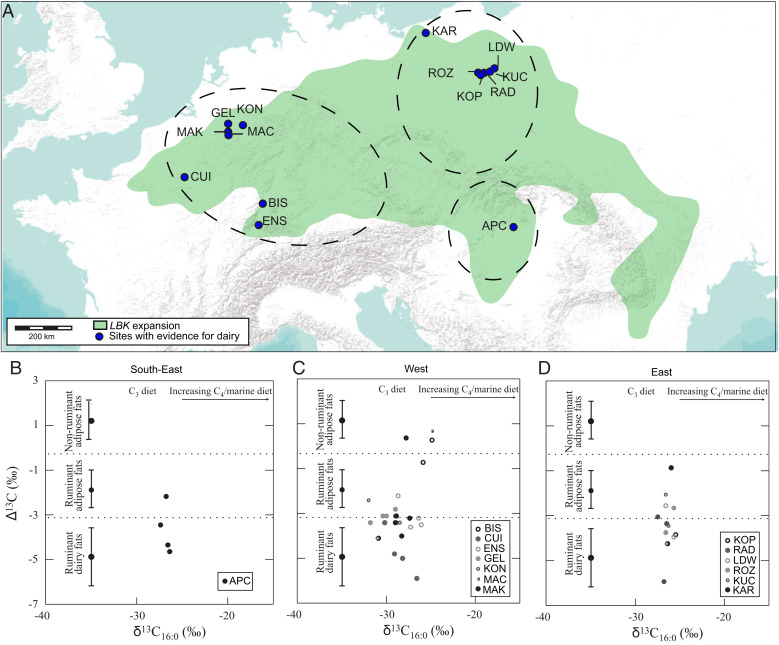
(*A*) Map of the LBK sites sampled in the southeastern, eastern, and western areas. (*B*–*D*) Δ^13^C (= δ^13^C_18:0_ to δ^13^C_16:0_) values plotted against the δ^13^C_16:0_ values of FAs extracted from the selected potsherds from the southeast, west, and east areas, respectively. Each dot corresponds to one vessel, and the ranges correspond to modern references (5, 13). APC, Apc-Bereklja 1; BIS, Bischoffsheim; CUI, Cuiry-lès-Chaudardes; ENS, Ensisheim; GEL, Geleen-Janskamperveld; KAR, Karwowo 2; KON, Königshoven; KOP, Kopydłowo 6; KUC, Kuczkowo 5; LDW, Ludwinowo 7; MAC, Maastricht-Cannerberg; MAK, Maastricht-Klinkers; RAD, Radojewice 29; and ROZ, Rożniaty 2.

Interestingly, at most sites, dairy residues were present in lower concentrations than adipose fats from ruminant and nonruminant animals. Hence, viable dairy residue candidates for direct dating were not available for all the sites where dairy fats were identified. These differences in concentration may suggest diverse food processing practices using pottery vessels. A total of 14 sites (30% of the sites where dairy fat was identified) were deemed suitable for radiocarbon dating: the emergent LBK region (Hungary, 1 site), the eastern area of extension (Poland, 6 sites) and the western area of extension (France, 3 sites; the Netherlands, 3 sites; Germany, 1 site; Fig. 1*A*). The pottery selection comprised a total of 43 vessels; 31 residues identified as dairy, 9 as ruminant adipose, and 4 as nonruminant adipose products (Fig. 1 *B*–*D*, Table 1, and *SI Appendix*, Table S1).

**Table 1.** Summary of dated potsherds with their context description, fat type, TLE concentration, compound-specific δ^13^C values, conventional radiocarbon ages, and X^2^ tests

Sample no.	Context	Fat type	Conc.	δ^13^C_16:0_	δ^13^C_18:0_	Δ^13^C	Aquatic markers	Lab. No.	Conv. ^14^C age (B.P.)	T’
			(μg/g)	(‰)	(‰)	(‰)				
APC-4182	Single sherd, context 1058, archaic phase	RAF	1,002	−26.9	−29.0	−2.0		BRAMS-2672	6,350 ± 25	
APC-4263	Single sherd, context 70, Zeliezovce phase	RDF	858	−27.4	−30.9	−3.5		BRAMS-2673	4,059 ± 24	
BIS-4527	Single sherd, context 538, Late LBK IVa1	RDF	2,477	−31.7	−35.8	−4.1		BRAMS-1705	6,087 ± 27	2.32
BIS-4529	Single sherd, context 538, Late LBK IVa1	RAF	2,021	−26.7	−27.4	−0.7		BRAMS-1209	6,139 ± 25	
BIS-4531	Single sherd, context 538, Late LBK IVa1	NRAF	973	−25.7	−25.4	0.3		BRAMS-1267	6,138 ± 36	
CUI-5708	Single sherd, context 25, Late LBK I	RDF	881	−26.6	−32.5	−5.9		BRAMS-1917	6,236 ± 27	
CUI-5801	Single sherd, context 386, Late LBK II	RDF	9,886	−28.2	−33.3	−5.0		BRAMS-2021	6,136 ± 25	
ENS-5913	Single sherd, context 9, Early LBK II	RDF	1,177	−28.1	−31.7	−3.6		BRAMS-1915	6,324 ± 26	0.43
ENS-5915	Single sherd, context 9, Early LBK II	RDF	771	−27.2	−30.4	−3.2		BRAMS-1916	6,348 ± 26	
ENS-5934	Single sherd, context 28, Early LBK II	RDF	1,647	−27.0	−30.5	−3.5		BRAMS-1958	6,270 ± 25	3.15
ENS-5940	Single sherd, context 28, Early LBK II	RAF	2,082	−29.5	−31.7	−2.2		BRAMS-2031	6,206 ± 26	
GEL-3298	Single sherd, context 49016, LBK II	RAF	577	−29.0	−31.8	−2.8	Y	BRAMS-2032	6,224 ± 25	
GEL-3299	Single sherd, context 49016, LBK II	RDF	2,743	−30.0	−33.1	−3.1	Y	BRAMS-1924	X	
GEL-3316	Single sherd, House 3, LBK Ib	RDF	523	−29.0	−32.2	−3.1		BRAMS-2611	6,154 ± 31	
KAR-3636	Single sherd, context 47 trench 2A, LBK III	NRAF	3,316	−26.0	−26.9	−0.9	Y	BRAMS-2028	6,204 ± 25	
KAR-3677	2 refitted sherds context 43, LBK III	RDF	1,900	−26.4	−30.7	−4.3	Y	BRAMS-2025	6,236 ± 26	
KON-5594	Single sherd, context 522, Jungere LBK, LBK III	RAF	531	−32.0	−34.4	−2.4		BRAMS-2029	6,276 ± 24	17.94
KON-5598	Single sherd, context 522, Jungere LBK	RDF	1,023	−31.0	−35.1	−4.1	Y	BRAMS-2026	6,123 ± 27	
KON-5617	Single sherd, context 522, Jungere LBK	RDF	678	−28.5	−31.9	−3.4		BRAMS-2023	X	
KUC-3751	Single sherd, context B228, LBK III	RDF	443	−26.3	−29.8	−3.5	Y	BRAMS-2669	X	
KUC-3755	Single sherd, context B228, LBK III	RDF	6,716	−26.5	−30.8	−4.3	Y	BRAMS-2603	6,189 ± 31	0.73
KUC-3763	Single sherd, context B228, LBK III	RAF	1,948	−26.6	−28.7	−2.1	Y	BRAMS-2604	6,227 ± 32	
LDW-2267	2 refitted sherds context A49, LBK IIB	RAF	323	−26.6	−29.2	−2.6		BRAMS-2024	6,177 ± 26	
LDW-2272	Single sherd, context A50, LBK IIB	RDF	1,628	−25.7	−29.7	−4.0		BRAMS-1919	6,252 ± 31	
MAC-3041	Single sherd, context 700	RDF	2,154	−28.8	−32.2	−3.4		BRAMS-2667	X	
MAC-3037	Single sherd, context 140	NRAF	4,328	−24.8	−24.1	0.7	Y	BRAMS-2668	6,353 ± 24	
MAK-3094	Single sherd, context 207, LBK IIb	RDF	494.0	−29.0	−32.4	−3.4		BRAMS-2022	X	
MAK-3102	Single sherd, context 22, LBK IIb	RDF	2,154	−28.8	−31.9	−3.1		BRAMS-2602	6,201 ± 36	
RAD-3814	Single sherd, context B75, LBK IIB	RDF	1,676	−27.5	−30.6	−3.1		BRAMS-2605	6,325 ± 31	0.2
RAD-3817	Single sherd, context B75, LBK IIB	RDF	4,650	−26.5	−29.9	−3.4	Y	BRAMS-2607	6,344 ± 29	
ROZ-3847	4 refitted sherds, context E36, LBK IIA	RDF	1,736	−26.6	−30.4	−3.8		BRAMS-2670	6,414 ± 24	
ROZ-3850	3 refitting sherds, context E50, LBK IIA	RAF	4,076	−25.7	−28.4	−2.7		BRAMS-2671.1	6,436 ± 24	136.59
								BRAMS-2671.2	6,005 ± 28	

See *SI Appendix*, Table S1 for details. RAF, ruminant adipose fats; RDF, ruminant dairy fats; and NRAF, nonruminant adipose fats. Y denotes the presence of at least one aquatic biomarkers (TMTD and >C_20_ APAAs), X potsherds which dating on individual FAs failed the internal quality control. Conc., concentration; Lab., laboratory; Conv., conventional.

### Radiocarbon Dates.

A total of 27 potsherds produced statistically consistent radiocarbon measurements on the C_16:0_ and C_18:0_ FAs (which were combined before calibration), 16 of which provide direct dates on dairy residues and 11 indirect dates from adipose residues (Table 1). A total of five potsherds failed our quality control criteria (i.e., results on the individual C_16:0_ and C_18:0_ FAs must not be significantly different at the 5% significance level), giving an 85% success rate. Unfortunately, 11 of the 43 selected potsherds did not yield sufficient C to provide radiocarbon measurements on both FAs, probably due to spatially inhomogeneous distribution of lipids in the potsherds (21, 26). Although we constrained sampling to potsherds with the highest concentrations of dairy fats, the overall lower concentration of dairy residues in potsherds compared to adipose residues explains 1) the proportion of potsherds with dairy/adipose residues successfully dated and 2) the failure to extract enough C for a radiocarbon date in 11 vessels.

In five of the six cases where dates have been obtained on more than one potsherd from a context, the measurements are statistically consistent (Table 1). This suggests that the sherds may be from closed assemblages and provides additional confidence in the reliability of the CSRA data. KON-5594, a single sherd from context 522 at Königshoven, Germany is significantly earlier than KON-5598 from the same deposit (typologically dated to the Late LBK phase) and is probably residual in this deposit.

The uncalibrated radiocarbon ages of the fat residues range from c. 6450 B.P. to c. 6000 B.P. The two oldest measurements were obtained from Poland which, when calibrated, predate the published modeled ^14^C date ranges for the earliest LBK features in the region and elsewhere (27). In the case of Rożniaty, the potsherds are refitting, typologically characteristic of early LBK and the LBK is the first culture with pottery in the area. This suggests that the radiocarbon ages from these sherds could be affected by a reservoir effect due to an aquatic contribution. One of the vessels (ROZ-3850) was also dated a second time with the new date about 400 y younger, suggesting that lipids in this potsherd are inhomogeneously distributed and likely containing both terrestrial and aquatic fats.

### Potential Reservoir Effects Considerations.

The LBK sites are usually located adjacent to large rivers or their tributaries ( e.g., Danube, Meuse, Rhine, and Vistula) and sometimes lakes, allowing access of LBK farmers to freshwater resources. In the light of the ^14^C dates obtained in this study, we investigated whether freshwater reservoir effects (FRE) are likely to influence the dating of potsherds through systematic screening of the all the lipid residues from each of the studied sites for aquatic biomarkers, including long-chain > C_20_ dihydroxy fatty acids (DHYAs), long-chain > C_20_ ω-(*o*-alkylphenyl)alkanoic acids (APAAs), and the 4,8,12-trimethyltridecanoic acid (TMTD) isoprenoid acid (28). We also considered the δ^13^C values of individual C_16:0_ and C_18:0_ FAs to further identify the potential presence of lipids from aquatic sources (28).

**Fig. 2.**
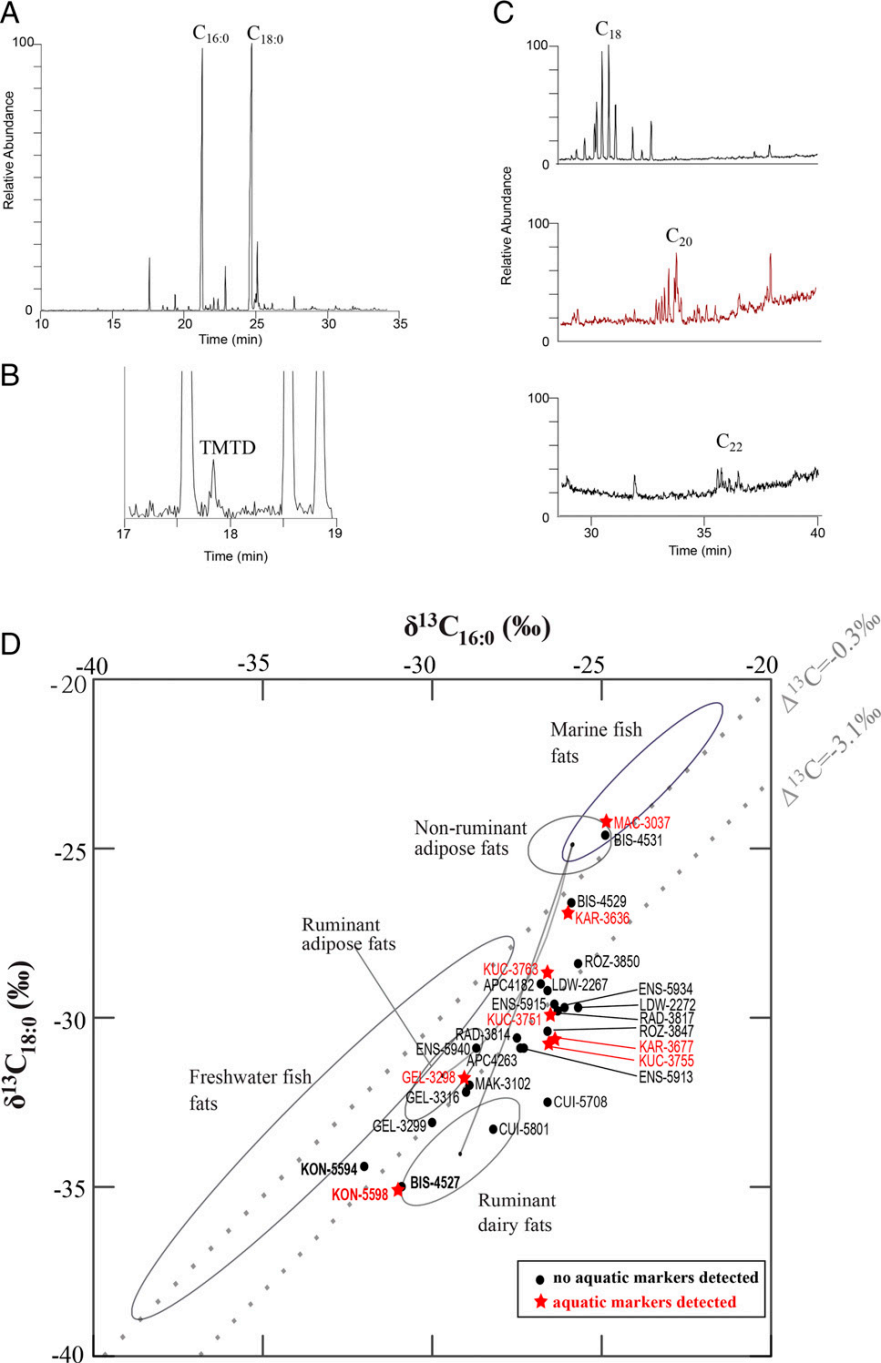
(*A*) Partial gas chromatogram of a potsherd from Rożniaty (ROZ-3835 not dated) dominated by the C_16:0_ and C_18:0_ FAs characteristic of degraded animal fats and exhibiting aquatic biomarkers (*B*) isoprenoid FAs and (*C*) long-chain APAAs. (*D*) δ^13^C_18:0_ values plotted against the δ^13^C_16:0_ values of FAs for the dated potsherds. Ellipses correspond to modern reference ranges of UK terrestrial animals (5), UK marine fish (28), and freshwater fish from Denmark, Kazakhstan, and the United Kingdom (28, 46).

Overall, no DHYAs biomarkers were recovered at the sites, but all of them, except the Alsatian sites (22), showed the presence of the TMTD biomarker in some of the TLEs (total lipid extracts). Both long-chain >C_20_ APAAs were found at the site of Rożniaty in 39% of the TLEs (excluding the dated potsherds) and in the only dated potsherd extract from the site of Maastricht-Cannerberg (the Netherlands; *SI Appendix*, Table S1 and Fig. 2 *A*–*C*). These APAAs form by thermally induced cyclization of unsaturated FAs when fats are heated in associated with ceramic fabric at high temperatures. Seven other sites contained only C_20_ APAAs, which is not exclusively of aquatic origin (29), together with the TMTD. The site of Rożniaty showed the highest recovery of aquatic biomarkers in the vessels (i.e., in 55% of the TLEs). This suggests that most potsherds at this site contained lipid residues derived from aquatic resources and would thus be affected by a reservoir effect, including the two dated potsherds where aquatic markers were not detected. Such reservoir effects affecting ^14^C dates even in the absence of aquatic biomarkers detection has been previously highlighted (26) and would be likely at sites with intensive processing of aquatic resources in pottery vessels, which is the case at the site of Rożniaty.

The δ^13^C values of individual C_16:0_ and C_18:0_ FAs of the dated potsherds are characteristic of ruminant dairy, ruminant adipose, and nonruminant adipose fats, with mixing of commodities in vessels being evident from intermediate δ^13^C values [Fig. 2*D* (30)], although for Polish sites, an environmental effect is more likely than for the western LBK sites, based on δ^13^C values of dairy residues recovered from sieves (7). Except for potsherd MAC-3037 at Maastricht-Cannerberg, where those δ^13^C values overlap with reference porcine and marine fats, there is no clear evidence for aquatic resource processing. There is also no correlation between the potsherds where aquatic markers were found and the δ^13^C values of individual C_16:0_ and C_18:0_ FAs that might suggest that these would have been mixed with terrestrial resources. The sites producing only the TMTD biomarker and lacking > C_20_ APAAs suggest minor freshwater resources processing in the pottery.

### Chronology of Early Dairying in the LBK.

**Fig. 3.**
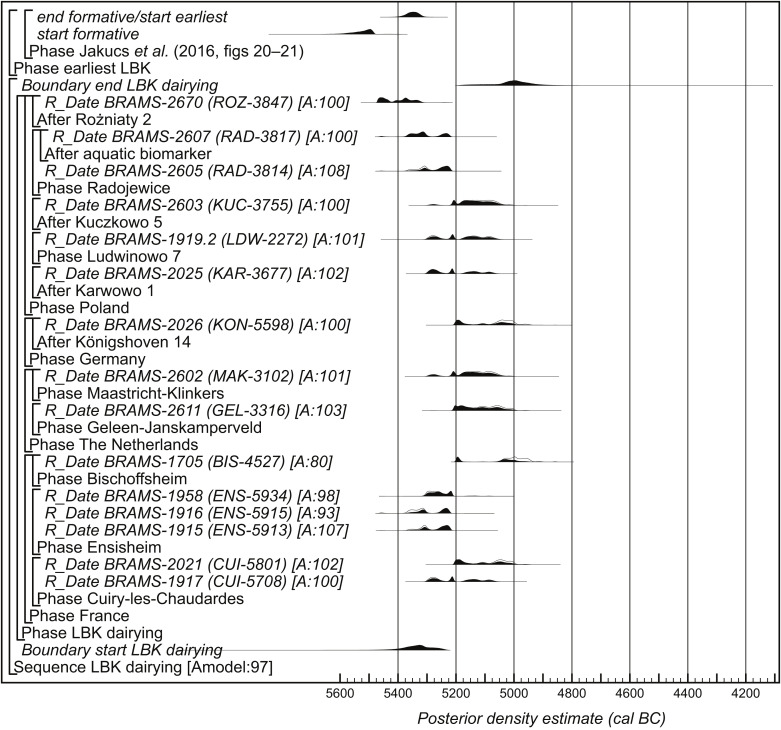
Probability distributions of CSRA dates from lipid residues diagnostic of dairying in LBK pottery vessels. Each distribution represents the relative probability that an event occurs at a particular time. For each of the dates, two distributions have been plotted: one in outline, which is the result of simple radiocarbon calibration, and a solid one, based on the chronological model used. Distributions other than those relating to particular samples correspond to aspects of the model. For example, the distribution “start LBK dairying” is the estimated date when dairying was introduced. The distribution followed by “?” has been excluded from the model. The large square brackets down the *Left* side of the figure, along with the OxCal keywords, define the overall model exactly.

Fig. 3 shows a model which estimates the date when dairying was introduced into the areas of Europe which used LBK ceramics. This includes only the CSRA dates on lipid residues that are diagnostic of dairying. Since there is a potential for reservoir effects (bias to older ages) in the ages of those in which aquatic biomarkers have been found, these are included as termini post quos as a conservative approach. As discussed in *SI Appendix*, section A, BRAMS-2670 (ROZ-3847), which is likely to have a reservoir effect, is also modeled as terminus post quem.

This model has good overall agreement (Amodel: 97) and suggests that dairying began in *5445*-*5230 cal BC (95% probability; start LBK dairying*; Fig. 3), probably in *5380*-*5275 cal BC (68% probability)*. This date estimate can be compared with that for the transition between the formative and earliest phases of LBK ceramics presented by Jakucs et al. (figures 20 and 21 in ref. 27). This is the time of the LBK expansion out of the Danube valley, which saw the first LBK occupation in many of the areas of eastern Europe covered by this study. Recalculated using IntCal20 (31), this occurred in *5395*-*5310 cal BC (95% probability; end formative/start earliest*; Fig. 3), probably in *5370*-*5325 cal BC (68% probability)*. This is clearly compatible with the date estimate for the start of dairying—indeed, the medians of these distributions differ by only 15 y.

An alternative model, which includes all the CSRA dates listed in Table 1, again has good overall agreement (Amodel: 101; *SI Appendix*, Fig. A). In this model, all the dates with aquatic biomarkers and BRAMS-2670 (ROZ-3847) have been included as termini post quos, as has BRAMS-2029 (KON-5594), which is considered residual (see *Radiocarbon Dates*). The two pairs of statistically consistent radiocarbon ages obtained from the group of sherds ROZ-3850 at Rożniaty 2 were found to be significantly different [T' = 136.6, T'(5%) = 3.8, ν = 1], and so they have been excluded from the analysis. Some of the samples included in this model are clearly less directly related to dairying than those in the previous model, although this is offset by the additional amount of data available and the consistency of results on dairy/nondairy lipids noted in *Radiocarbon Dates*. Further discussion of this model is provided in *SI Appendix*, section A.

The alternative model provides a very similar estimate for the start of dairying in the LBK in *5415-5240* *cal BC* (95% probability; start LBK dairying; *SI Appendix*, Fig. A), probably in *5385-5300* cal BC (64% probability) or *5275-5260* *cal BC* (4% probability), which is again entirely compatible with the date estimate for transition between the formative and earliest phases of LBK ceramics (the medians of these distributions differ by 14 y).

The greater number of dates in this model enables the investigation of potential regional trends in the adoption of dairying across the LBK oecumene, although these remain provisional. For this analysis, comparisons were made with the date estimates for the beginning of the earliest LBK in the regions defined by Jakucs et al. (figures 22 and 23 in ref. 27) [i.e., southeastern groups are located south and East of the Danube (Hungary), the eastern groups are located north of the Danube and east of Linz (Austria), and the western groups are upstream of the Danube and in the Rhine (Germany) and Meuse valley (Netherlands)].

**Fig. 4.**
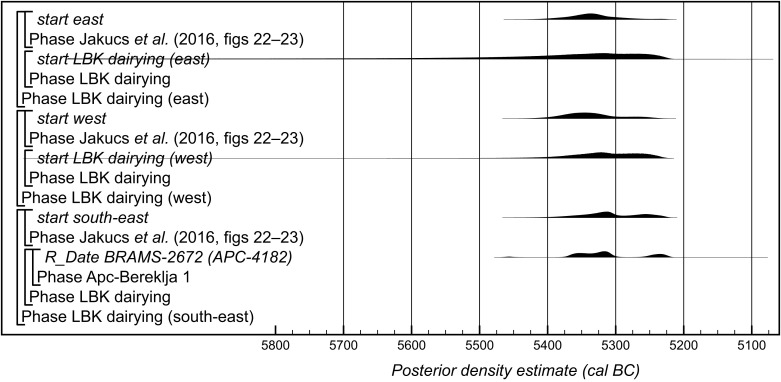
Probability distributions key parameters for the start of dairying and the appearance of the earliest LBK pottery in different regions of Europe, derived from the models defined in *SI Appendix*, Fig. A and in Jakucs et al. (figures 22 and 23 in ref. 25), recalculated using IntCal20.

In the southeast area, only the site of Apc-Bereklja 1 (Hungary) yielded material suitable for compound-specific lipid ^14^C dating (Table 1). The ^14^C date for APC-4263 (BRAMS-2673) is clearly too recent for an LBK potsherd and is likely intrusive from a later culture. BRAMS-2672 (APC-4182), determined to represent ruminant adipose lipids without any aquatic biomarkers, provides an indirect ^14^C date for early dairying supported by other sherds from the archaic phase at the site. It is statistically consistent with the only other measurement available from this phase at Apc-Bereklja 1 (OxA-25187, a disarticulated cattle bone, 6290 ± 40 B.P.; T' = 1.6, T'(5%) = 3.8, ν = 1) and clearly compatible with the date estimate for the start of the earliest LBK in the southeast region of Jakucs et al. (figures 22 and 23 in ref. 27), as the medians of the distributions vary by just 10 y (Fig. 4). This site, producing some of the oldest LBK ^14^C dates in Hungary, is nonetheless not the earliest LBK settlement in the region based on typological criteria and stone tools analyses (32).

In the western area, lipids from 15 vessels at seven sites have been ^14^C dated. Of these, three contained aquatic biomarkers and BRAMS-2029 (KON-5594 at Königshoven, Germany, considered residual based on comparison with the other dated sherd from the context) were incorporated in the model as termini post quos. This model suggests that dairying was introduced to the western area of this study in *5420*-*5225 cal BC (95% probability; start LBK dairying (west)*; *SI Appendix*, Fig. C), probably in *5355*-*5250 cal BC (68% probability)*. This may be (*64% probability*) a little later than the date estimate for the start of the earliest LBK in the western region of Jakucs et al. (figures 22 and 23 in ref. 27), as the medians of these distributions differ by 27 y (Fig. 4). This does not reflect a lag in the adoption of dairying by LBK communities in this area but rather differences in the regions considered in the two studies. Jakucs et al. (figure 1 in ref. 27) considered the earliest LBK pottery only, and so their western region was concentrated in the area to the east of the upper Rhine valley (Germany); in contrast, the samples dated as part of this study come from the area west of the Rhine (France, the Netherlands, Germany), which may have seen LBK colonization slightly later.

In the eastern area, 10 vessels from five sites in the Polish Lowlands have been ^14^C dated. Of these, five contained aquatic markers (Table 1). The two dated potsherds from Rożniaty produced neither aquatic markers nor significant freshwater or marine contributions based on their δ^13^C_16:0_ and δ^13^C_18:0_ values (Fig. 2*D*). However, the two statistically consistent pairs of measurements on two different TLEs from ROZ-3850 suggest high spatial heterogeneity of lipids in this vessel and that a high contribution of aquatic resources at the site could have been missed in the particular TLE used for these analyses. Given the sensitivity of the model outputs to the inclusion of BRAMS-2670 (ROZ-3847) in the analysis (*SI Appendix*, section A), the presence of a reservoir effect in this potsherd must be suspected, and hence the date was included in the model as a terminus post quem. The prevalence of aquatic components in the lipid extracts from the Polish Lowlands means that only three dates are fully included in the model for LBK dairying in the eastern region, and so the estimate for its beginning in this area is imprecise (*SI Appendix*, Fig. C). It is clearly, however, compatible with the date estimate for the start of the earliest LBK in the eastern area provided by Jakucs et al. (figures 22 and 23 in ref. 27; Fig. 4). Again, it should be noted that the eastern regions covered by the two studies differ substantially (cf. Fig. 1 and figure 1 in ref. 27).

## Discussion

Dairy exploitation was present during the LBK, although unevenly distributed among the sites and regions and not necessarily at a large scale (22, 25). The overall low lipid concentration of dairy residues found in LBK pottery, particularly at the Hungarian sites, may also reflect the intensity for which milk was processed in the pottery vessels compared to animal carcass products. This “low” intensity of milk processing has therefore impacted the number of potsherds datable across the sites.

Interestingly, aquatic resource processing in LBK pottery was highlighted through ^14^C dating and included residues identified as dairy. Aquatic remains are usually scarce at LBK sites, even where intense sieving was performed (33), suggesting these resources were only exploited on a small scale. Exploitation of aquatic resources was particularly important and impacting the ^14^C dates at the site of Rożniaty 2 (Poland), which is one of the oldest known sites of the Kuyavia region of Poland. This dataset suggests that despite a subsistence largely based on the primary and secondary products of domesticated animals, a low-level exploitation of aquatic resources continued during the LBK.

The spatial nonuniformity of dairy use during the LBK raises questions regarding the spread and adoption of dairy practices among LBK farmers and whether these were related to the several migration waves of LBK farmers with diverse animal management practices or were influenced by other populations in certain areas.

Jakucs et al. (27) dated the appearance of the earliest LBK settlements in the 54th century BC. These estimates are based on ^14^C dates from sites presenting the earliest LBK typologies of the Meier-Arendt chronology, which have not been identified in the sites we targeted for ^14^C dating in this study. Our ^14^C dates nonetheless suggest that dairying was practiced within the earliest settlements in the several areas of expansion and were not gradually adopted through the ages.

The southeast area corresponds to the emergent region of the LBK and most Hungarian sites, exhibited evidence of a dairy economy. The region could have been largely influenced by the dairy economy of the Balkans, which dates back to the 6th millennium BC (11).

The western area of expansion resulted from several migration waves especially in Alsace (33, 34). The farmers in the Lower Alsace likely arrived from the Neckar Valley and those of the Upper Alsace from the Danube region. While the milk signal was intense in pottery from the Upper Alsace (Ensisheim, France) from the early LBK phase, the only evidence of dairy in Lower Alsace dates from the Late LBK phase IVa1 at Bischoffsheim (France). This suggests that dairying was not an intensive part of the economy in the Lower Alsace in contrast to Middle Neolithic culture (22). Alsatian populations later migrated northwest to the Seine basin where the LBK site of Cuiry-lès-Chaudardes (Aisne Valley, France) also showed intense dairy practices (6). Dutch sites possibly arise from a migration wave coming from Lower Rhine (35, 36). The Dutch sites of Maastricht-Klinkers, Geleen-Janskamperveld, and the German site of Königshoven provided contemporaneous ^14^C ages, suggesting the synchronicity of possible links between the settlements in the northwest area. In the western LBK sites (France and the Netherlands), the pottery styles of La Hoguette and Limburg with decorations presenting some Mediterranean characteristics similar to Impressed Ware/Cardial groups ceramics are commonly recovered within LBK assemblages (37–40). The Mediterranean cultural groups presented an economy based on dairy as early as the first half of the 6th millennium BC and have been hypothesized to have migrated northwards following the Rhône–Rhine axis, which could have influenced the adoption of dairy by LBK farmers in this area (8, 40).

In the eastern area, Polish settlements may have resulted from several migration waves from the Carpathians relating to Hungarian migration waves and/or the Elbe river catchment of Central Europe (41, 42). Particularly, the origins of the LBK settlements in the Kuyavia region are not well known (41, 42). The chronology of the Polish Lowlands is still debated especially the earliest (älteste) phase I (43–45), but following phase from the early LBK IIA is better recognized and includes the sites of Rożniaty 2, Radojewice 29, and Ludwinowo 7 (43, 44). The Pyrzyce region in northwest Poland was less densely occupied, and the site of Karwowo (Middle LBK) is one of the oldest sites known in the western side of the region to this date. In the eastern area, populations from the Anatolia and the Balkans, where the exploitation of dairy products dates back to the 7th to 6th millennium BC, could also have influenced the adoption of dairying in this area (6).

## Conclusions

Despite some sites possibly being affected by FREs, the direct ^14^C dates of FAs from food residues from pottery support the adoption of dairying in different sites and regions arriving with the first settlers in those areas, and the practice was not gradually adopted through the LBK phases. Therefore, dairying practices in Central Europe are likely as old as the LBK culture itself. Interestingly, dairying was not uniformly spatially distributed within the LBK culture, suggesting either influence by migration waves from LBK farmers with various animal management practices or might originate from influences from other cultural groups in nearby areas (e.g., Mediterranean influence in the western area). Further insight into LBK migration waves, together with lipid residue analyses and direct dating of pottery from groups preceding or being in contact with the LBK farmers will shed more light on the precise timing of the adoption and spread of dairy practices in the southeastern, eastern, and western regions of Central Europe.

## Methods

### Animal Fat Discrimination.

Screening for aquatic markers present in low abundances in TLEs was performed following established procedures (28). The δ^13^C_16:0_ and δ^13^C_18:0_ values were determined on all TLEs containing animal fats to distinguish their sources. We used Δ^13^C values below −3.1‰ to identify dairy residues, as this has been shown to be a robust proxy in a wide range of environments (5, 7, 14). For the studied potsherds (especially those presenting aquatic markers), we used the δ^13^C_16:0_ and δ^13^C_18:0_ values on the TLEs, δ^13^C_16:0_ and δ^13^C_18:0_ modern reference values of terrestrial and aquatic organisms (5, 28, 46), and mixing curves (30).

### CSRA of Pot Lipids.

The potsherds were cleaned with a modeling drill and crushed into fine powder (2 to 10 g of clay depending on lipid concentrations). Lipids were extracted from the clay using a methanol/sulphuric acid extraction (4% *v/*v, 70 °C, 1 h, 3 × 8 mL, mixed every 10 min). The liquid fractions were centrifuged in culture tubes, then the three acidic supernatants combined in a second culture tube with 5 mL Milli Q-Water. Lipids were extracted by performing a liquid/liquid extraction with 4 × 5 mL *n*-hexane (21).

Isolation of specific compounds was performed on a Hewlett Packard gas chromatograph (GC) interfaced to a Gerstel Preparative Fraction Collector. Compounds were isolated 40 times into individual solventless traps based on their retention times (20). Compounds were recovered from the traps by transferring the glass wool into Al capsules, then combusted in an Elementar Isotope Cube elemental analyzer, and the resulting CO_2_ transferred to an IonPlus AGE 3 graphitization system for their reduction to graphite for analysis by accelerator mass spectrometry (AMS) at the Bristol Radiocarbon Accelerator Mass Spectrometry (BRAMS) facility (20, 47).

### Calibration and Modeling.

The C_16:0_ and C_18:0_ FA ^14^C dates obtained from the same potsherds (where statistically indistinguishable at the 5% significance level) were combined as described in Casanova et al. (21) to provide conventional radiocarbon ages on pottery vessels. The chronological modeling was performed using OxCal version 4.4.3 ( 48) and the currently internationally agreed radiocarbon calibration curve for the northern hemisphere, IntCal20 (31). Highest posterior density intervals of posterior distributions are cited in italics. All models are defined by the CQL2 code provided in the *SI Appendix*.

## Supplementary Material

Supplementary File

## Data Availability

Previously published data were used for this work (21). All other study data are included in the article and/or *SI Appendix*.
