# Supplementary material for: Dating the emergence of dairying by the first farmers of Central Europe using 14C analysis of fatty acids preserved in pottery vessels
Source: Proc Natl Acad Sci U S A. 2022 Oct 17;119(43):e2109325118. doi: 10.1073/pnas.2109325118 (PMC9618069; doi:10.1073/pnas.2109325118)

Supplementary information

Emergence of Dairying in the 6th millennium BC Amongst the *Linearbandkeramik* Farmers of Central Europe Directly ^{14}C-dated Through Fat Residues Preserved in Pottery Vessels

Emmanuelle Casanova[1], Timothy D.J. Knowles[1,2], Alex Bayliss[3], Mélanie Roffet-Salque[1], Volker Heyd[4], Joanna Pyzel[5], Eric Claβen[6], László Domboróczki[7], Michael Ilett[8], Philippe Lefranc[9], Christian Jeunesse[10], Arkadiusz Marciniak[11], Ivo van Wijk[12] and Richard P. Evershed[1,2]

[1]Organic Geochemistry Unit, School of Chemistry, Cantock's Close, Bristol, BS8 1TS, UK

[2]Bristol Radiocarbon Accelerator Mass Spectrometry Facility, University of Bristol, 43 Woodland Road, Bristol, BS8 1UU, UK

[3]Scientific Dating, Historic England, Cannon Bridge House, 25 Dowgate Hill, London, EC4R 2YA, UK

[4] Department of Cultures / Archaeology, University of Helsinki, Unioninkatu 38, 00014, Finland

[5]Institute of Archaeology and Ethnology, University of Gdansk, ul. Bielanska 5, PL 80-851 Gdańsk, Poland

[6]LVR-State Service for Archaeological Heritage, Endenicher Str. 133, 53115 Bonn, Germany

[7]Dobó István Castle Museum, Var utca, Eger H-3300, Hungary

[8] UMR 8215 TRAJECTOIRES, Université Paris 1 Panthéon-Sorbonne, 21 allée de l'université, 92023 Nanterre, France

[9] UMR 7044/Institut national des recherches archéologiques préventives, University of Strasbourg , 10 rue Altkirch, 67100 Strasbourg, France

[10]U UMR7044, Misha, University of Strasbourg, 5 allé du général Rouvillois, 67083 Strasbourg, France

[11] Institute of Archaeology, Adam Mickiewicz University, 61-614 Poznan, Poland

[12] Archaeological research Leiden, Leiden University, Einsteinweg 2, 2333 CC Leiden, The Netherlands

Table S1: Details of selected and ^{14}C dated potsherds with their context description, fat type, concentration, compound-specific $\delta^{13}C$ values, conventional radiocarbon ages and X^2 tests. RAF = ruminant adipose fats, RDF = ruminant dairy fats, NRAF = non-ruminant adipose fats. X denotes potsherds which dating on individual fatty acids failed the internal quality control.

Sites	Sample #	Details	C (ug/g)	Fat type	$\delta^{13}C_{16:0}$ (‰)	$\delta^{13}C_{18:0}$ (‰)	$\Delta^{13}C$ (‰)	Aquatic marker	BRAMS#	Compound	Age (BP)	combined	T'	T' context
Apc-Bereklja 1, Hungary	APC-4217	Single sherd, context 697 Archaic phase	653	RDF	-26.6	-31.0	-4.4	-	BRAMS-1925	$C_{16:0}C_{18:0}$	-			
	APC-4200	Single sherd, context 589, Archaic phase	590	RDF	-26.4	-31.1	-4.7	-	-		-			
	APC-4182	Single sherd, context 1058, archaic phase	1002	RAF	-26.9	-29.0	-2.0	-	BRAMS-2672	$C_{16:0}$	6331 ± 29	6350 ± 25	0.7	
										$C_{18:0}$	6365 ± 28			
	APC-4263	Single sherd, context 70, Zeliezovce phase	858	RDF	-27.4	-30.9	-3.5	-	BRAMS-2673	$C_{16:0}$	4029 ± 29	4059 ± 24	2	
										$C_{18:0}$	4087 ± 28			
Bischoffsheim “AFUA du Stade”, Lower Alsace, France	BIS-4527	Single sherd, context 538, Late LBK IVa1	2477	RDF	-31.7	-35.8	-4.1	-	BRAMS-1705	$C_{16:0}$	6084 ± 32	6087± 27	0.2	2.32
										$C_{18:0}$	6090 ± 31			
	BIS-4529	Single sherd, context 538, Late LBK IVa1	2021	RAF	-26.7	-27.4	-0.7	-	BRAMS-1209	$C_{16:0}$	6177 ± 31	6139 ± 25	2.9	
										$C_{18:0}$	6103 ± 30			
	BIS-4531	Single sherd, context 538, Late LBK IVa1	973	NRAF	-25.7	-25.4	0.3	-	BRAMS-1267	$C_{16:0}$	6185 ± 57	6138 ± 36	1	
										$C_{18:0}$	6112 ± 44			
Cuiry-lès-Chaudardes, Aisne Valley, France	CUI-5708	Single sherd, context 25, Late LBK I	881	RDF	-26.6	-32.5	-5.9	-	BRAMS-1917	$C_{16:0}$	6252 ± 34	6236 ± 27	0.5	
										$C_{18:0}$	6218 ± 36			
	CUI-5776	Single sherd, context 378, Late LBK II	3531	RDF	-30.2	-33.6	-3.4	-	BRAMS-2020	$C_{16:0}$ $C_{18:0}$	6142 ± 32			
	CUI-5801	Single sherd, context 386, Late LBK II	9886	RDF	-28.2	-33.3	-5.0	-	BRAMS-2021	$C_{16:0}$	6138 ± 30	6136 ± 25	0	
										$C_{18:0}$	6134 ± 30			
	CUI-5735	Single sherd, context 241, Late LBK III	3417	RDF	-29.1	-33.9	-4.8	-	BRAMS-1918	$C_{16:0}$	6138 ± 37			
										$C_{18:0}$	-			
Ensisheim “Ratfeld”, Upper Alsace, France	ENS-5913	Single sherd, context 9, Early LBK II	1177	RDF	-28.1	-31.7	-3.6	-	BRAMS-1915	$C_{16:0}$	6345 ± 31	6324 ± 26	0.9	0.43
										$C_{18:0}$	6303 ± 31			
	ENS-5915	Single sherd, context 9, Early LBK	771	RDF	-27.2	-30.4	-3.2	-	BRAMS-1916	$C_{16:0}$	6383 ± 32	6348 ± 26	2.3	
										$C_{18:0}$	6314 ± 33			

		II												
	ENS-5934	Single sherd, context 28, Early LBK II	1647	RDF	-27.0	-30.5	-3.5	-	BRAMS-1958	$C_{16:0}$	6282 ± 30	6270 ± 25	0.3	3.15
										$C_{18:0}$	6258 ± 30			
	ENS-5940	Single sherd, context 28, Early LBK II	2082	RAF	-29.5	-31.7	-2.2	-	BRAMS-2031	$C_{16:0}$	6162 ± 33	6206 ± 26	3	
										$C_{18:0}$	6239 ± 30			
Geleen-Jankampersvel, Gratheide, The Netherlands	GEL-3271	Single sherd, context 49015, LBK I	1260	RDF	-30.4	-33.5	-3.1	TMTD	BRAMS-2027	$C_{16:0}$	6500 ± 33			
										$C_{18:0}$	-			
	GEL-3276	Single sherd, context 49015, LBK I	339	RDF	-31.8	-35.2	-3.4	-	BRAMS-1923	$C_{16:0}$ $C_{18:0}$	6142 ± 33			
	GEL-3298	Single sherd, context 49016, LBK II	577	RAF	-29.0	-31.8	-2.8	TMTD C20, C22? APAA	BRAMS-2032	$C_{16:0}$	6188 ± 31	6224 ± 25	2.3	
										$C_{18:0}$	6253 ± 29			
	GEL-3299	Single sherd, context 49016, LBK II	2743	RDF	-30.0	-33.1	-3.1	TMTD	BRAMS-1924	$C_{16:0}$	6304 ± 32	X	10.2	
										$C_{18:0}$	6444 ± 30			
	GEL-3316	Single sherd, House 3, LBK Ib	523	RDF	-29.0	-32.2	-3.1	-	BRAMS-2611	$C_{16:0}$	6110 ± 42	6154 ± 31	2	
										$C_{18:0}$	6191 ± 39			
Karwowo 2, Pomeria, Poland	KAR-3636	Single sherd, context 47 trench 2A, LBK III	3316	NRAF	-26.0	-26.9	-0.9	TMTD	BRAMS-2028	$C_{16:0}$	6176 ± 30	6204 ± 25	1.6	
										$C_{18:0}$	6230 ± 30			
	KAR-3677	2 refitted sherds context 43, LBK III	1900	RDF	-26.4	-30.7	-4.3	TMTD	BRAMS-2025	$C_{16:0}$	6255 ± 30	6236 ± 26	0.9	
										$C_{18:0}$	6214 ± 32			
Königshoven, Germany	KON-5594	Single sherd, context 522, Jungere LBK, LBK III	531	RAF	-32.0	-34.4	-2.4	-	BRAMS-2029	$C_{16:0}$	6253 ± 29	6276 ± 24	1.2	17.94
										$C_{18:0}$	6298 ± 29			
	KON-5598	Single sherd, context 522, Jungere LBK	1023	RDF	-31.0	-35.1	-4.1	TMTD	BRAMS-2026	$C_{16:0}$	6106 ± 34	6123 ± 27	0.5	
										$C_{18:0}$	6139 ± 34			
	KON-5617	Single sherd, context 522, Jungere LBK	678	RDF	-28.5	-31.9	-3.4	-	BRAMS-2023	$C_{16:0}$	6078 ± 39	X	7.7	
										$C_{18:0}$	6213 ± 30			
Kopydłowo, Kuyavia, Poland	KOP-2949	2 refitted sherds, context 25 B	548	RDF	-25.5	-29.4	-3.9		BRAMS-1920	$C_{16:0}$	-			
Kuczkowo 5, Kuyavia Poland	KUC-3751	Single sherd, context B228, LBK III	443	RDF	-26.3	-29.8	-3.5	TMTD	BRAMS-2669	$C_{18:0}$	6023 ± 28			
										$C_{16:0}$	6038 ± 29	X	45.5	
										$C_{18:0}$	6311 ± 28			
	KUC-3755	Single sherd, context B228, LBK III	6716	RDF	-26.5	-30.8	-4.3	TMTD	BRAMS-2603	$C_{16:0}$	6168 ± 42	6189 ± 31	0.4	0.73
										$C_{18:0}$	6206 ± 39			
	KUC-3763	Single sherd,	1948	RAF	-26.6	-28.7	-2.1	TMTD	BRAMS-2604	$C_{16:0}$	6206 ± 42	6227 ± 32	0.7	

		context B228, LBK III								$C_{18:0}$	6253 ± 43			
Ludwinowo 7, Kuyavia, Poland	LDW-2267	2 refitted sherds context A49, LBK IIB	323	RAF	-26.6	-29.2	-2.6	-	BRAMS-2024	$C_{16:0}$	6173 ± 36	6177 ± 26	0	
										$C_{18:0}$	6179 ± 30			
	LDW-2272	Single sherd, context A50, LBK IIB	1628	RDF	-25.7	-29.7	-4.0		BRAMS-1919.1	$C_{16:0}$	6150 ± 33	X	8.3	
										$C_{18:0}$	6283 ± 32			
								-	BRAMS-1919.2	$C_{16:0}$	6226 ± 42	6252 ± 31	0.7	
										$C_{18:0}$	6274 ± 39			
Maastricht Cannarberg, Limburg, The Netherlands	MAC-3041	Single sherd, context 700	2154	RDF	-28.8	-32.2	-3.4	-	BRAMS-2667	$C_{16:0}$	5851 ± 34	X	73.2	
										$C_{18:0}$	6274 ± 36			
	MAC-3037	single sherd	4328	NRAF	-24.8	-24.1	0.7	C20,C22 APAA	BRAMS-2668	$C_{16:0}$	6356 ± 29	6353 ± 24	0	
										$C_{18:0}$	6351 ± 28			
Maastricht Klinkers, Limburg, The Netherlands	MAK-3094	Single sherd, context 207, LBK IIb	494	RDF	-29.0	-32.4	-3.4	-	BRAMS-2022	$C_{16:0}$	6002 ± 30	X	35.6	
										$C_{18:0}$	6251 ± 29			
	MAK-3099	Single sherd, context 207, LBK IIb	7672	NRAF	-27.8	-27.5	0.4	TMTD	BRAMS-1922	$C_{16:0}C_{18:0}$	6300 ± 37			
	MAK-3102	Single sherd, context 22, LBK IIb	2154	RDF	-28.8	-31.9	-3.1	-	BRAMS-2602	$C_{16:0}$	6245 ± 61	6201 ± 36	0.7	
										$C_{18:0}$	6182 ± 42			
	MAK-3093	Single sherd, context 207, LBK IIb	1304	RDF	-28.3	-32.3	-4.0	-						
	MAK-3152	Single sherd, context 207, LBK IIb	820	RDF	-27.4	-30.6	-3.2	-						
Radojewice 29, Kuyavia, Poland	RAD-3813	Single sherd, context B75, LBK IIB	1022	RDF	-26.8	-32.8	-6.0	-	BRAMS-2606	$C_{16:0}$	6186 ± 37	-	4.6	
										$C_{18:0}$	6297 ± 37			
	RAD-3814	Single sherd, context B75, LBK IIB	1676	RDF	-27.5	-30.6	-3.1	-	BRAMS-2605	$C_{16:0}$	6360 ± 41	6325 ± 31	1.5	0.2
										$C_{18:0}$	6289 ± 41			
	RAD-3817	Single sherd, context B75, LBK IIB	4650	RDF	-26.5	-29.9	-3.4	TMTD	BRAMS-2607	$C_{16:0}$	6347 ± 37	6344 ± 29	0	
										$C_{18:0}$	6340 ± 39			
Rożniaty 2, kuyavia, Poland	ROZ-3847	4 refitted sherds, context E36, LBK IIA	1736	RDF	-26.6	-30.4	-3.8	-	BRAMS-2670	$C_{16:0}$	6373 ± 29	6414 ± 24	3.8	
										$C_{18:0}$	6452 ± 28			
	ROZ-3850	3 refitting sherds, context E50, LBK IIA	4076	RAF	-25.7	-28.4	-2.7	-	BRAMS-2671.1	$C_{16:0}$	6420 ± 29	6436 ± 24	0.6	136.6
										$C_{18:0}$	6450 ± 28			
									BRAMS-2671.2	$C_{16:0}$	5942 ± 42	6005 ± 28	1.0	
										$C_{18:0}$	6032 ± 32			

SI Section A

The alternative model for the chronology of dairying in the LBK world is shown in Figure A. This includes both dates on lipid extracts diagnostic of dairying and those diagnostic of other fat sources. Dates from residues that have aquatic biomarkers have been included as *termini post quos*, as they may have reservoir effects. KON-5594, a single sherd from context 522 at Königshoven, Germany is significantly earlier than KON-5598 from the same deposit (T′=17.9, T′(5%)=3.8, df=1) and is probably residual. In all the other cases where more than one vessel has been dated, the results are statistically consistent (Table AA), which suggests that the dated features contained closed assemblages of ceramics. This means that it is reasonable to use the CSRA dates on samples that are not diagnostic of dairying to estimate the date when dairying began as the association between the dated material and the activity is likely to be robust.

Two pairs of, statistically consistent, radiocarbon ages have been obtained from sherd group ROZ-3847 at Rożniaty 2, although as the two resultant ages are widely divergent (T′=136.6, T′(5%)=3.8, df=1), they have been excluded from the model. This difference may be explained by extreme inhomogeneity of lipids partitioning within this vessel, and the earlier dating of BRAMS-2671.1 may suggest the presence of fats derived from aquatic sources with a significant reservoir age in this part of the pot. If this is the case, this means that at Rożniaty 2, they may be reservoir effects in the CSRA dates without the detection of diagnostic aquatic biomarkers in the dated residue.

For this reason, the potential for a reservoir effect in BRAMS-2670 (ROZ-3847), which also did not provide aquatic biomarkers, has to be considered. Figure B shows a variant of the model illustrated in Figure 3 in which this date is fully included in the model (as if it had no reservoir effect). This model has good overall agreement (Amodel: 94), and BRAMS-2670 has good individual agreement (A: 79). The estimate for the start of dairying in the LBK provided by this model is substantially earlier than the equivalent distribution from the model shown in Figure 3 (the median is 65 years earlier). That a single measurement should have such a large effect on the model outputs must raise suspicion that this sample too contained a component of aquatic-derived lipids. For this reason, BRAMS-2670 has been included as a *terminus post quem* in the models presented here.

Figure C illustrates two models for the chronology of dairying in the eastern and western areas of the LBK distribution illustrated in Figure 1. These models incorporate the same approaches to modelling each date as the alternative model illustrated in Figure A.

Figure A: Probability distributions of CSRA dates from lipid extracts from LBK pottery. The format is identical to that of Figure 3. The large square brackets down the left-hand side of the diagram, along with the OxCal keywords, define the overall model exactly.

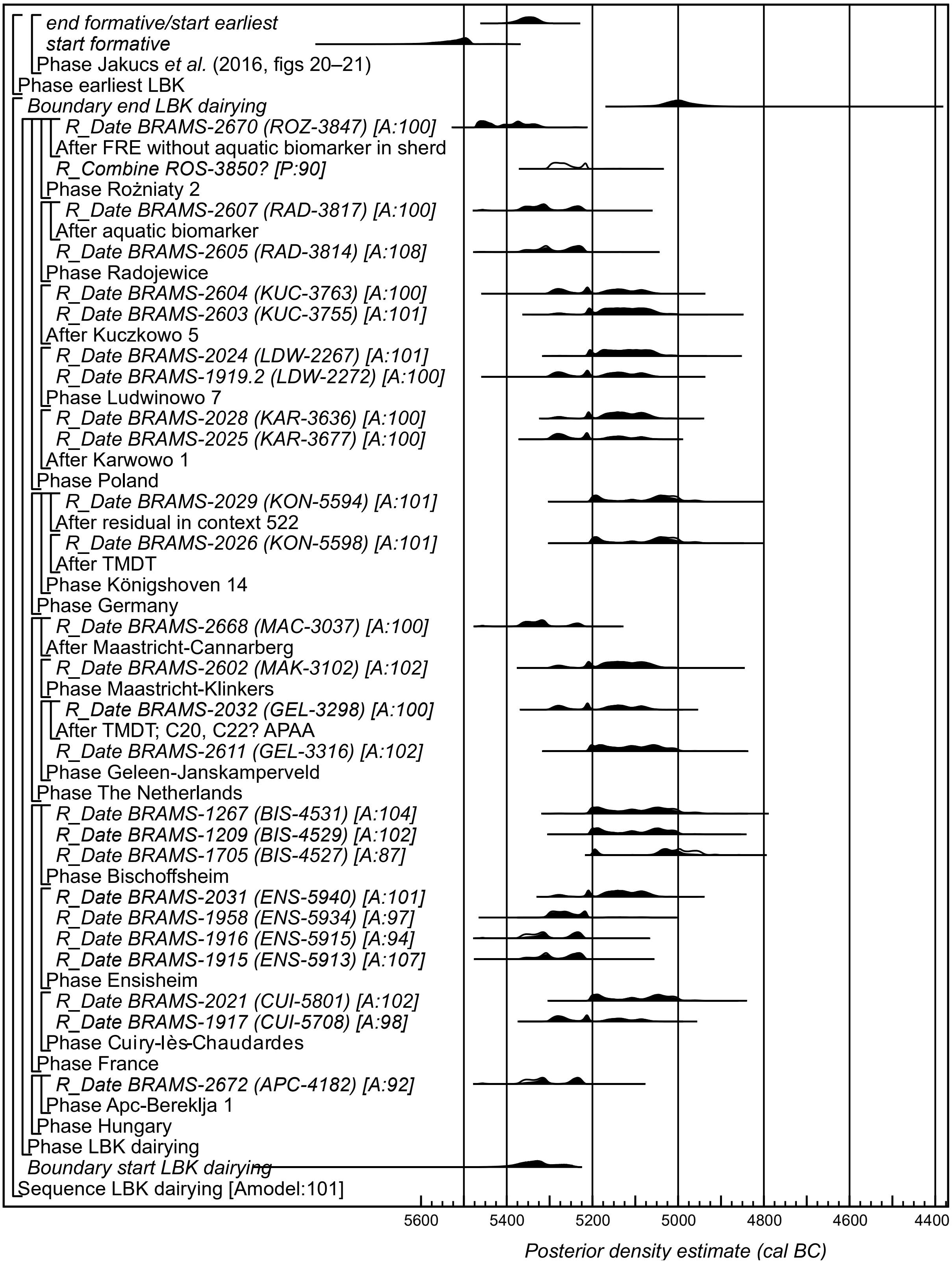

Figure B: Probability distributions of CSRA dates from lipid extracts diagnostic of dairying from LBK pottery, in which the lipid extract from ROZ-3847 is interpreted as deriving from fully terrestrial sources. The format is identical to that of Figure 3. The large square brackets down the left-hand side of the diagram, along with the OxCal keywords, define the overall model exactly.

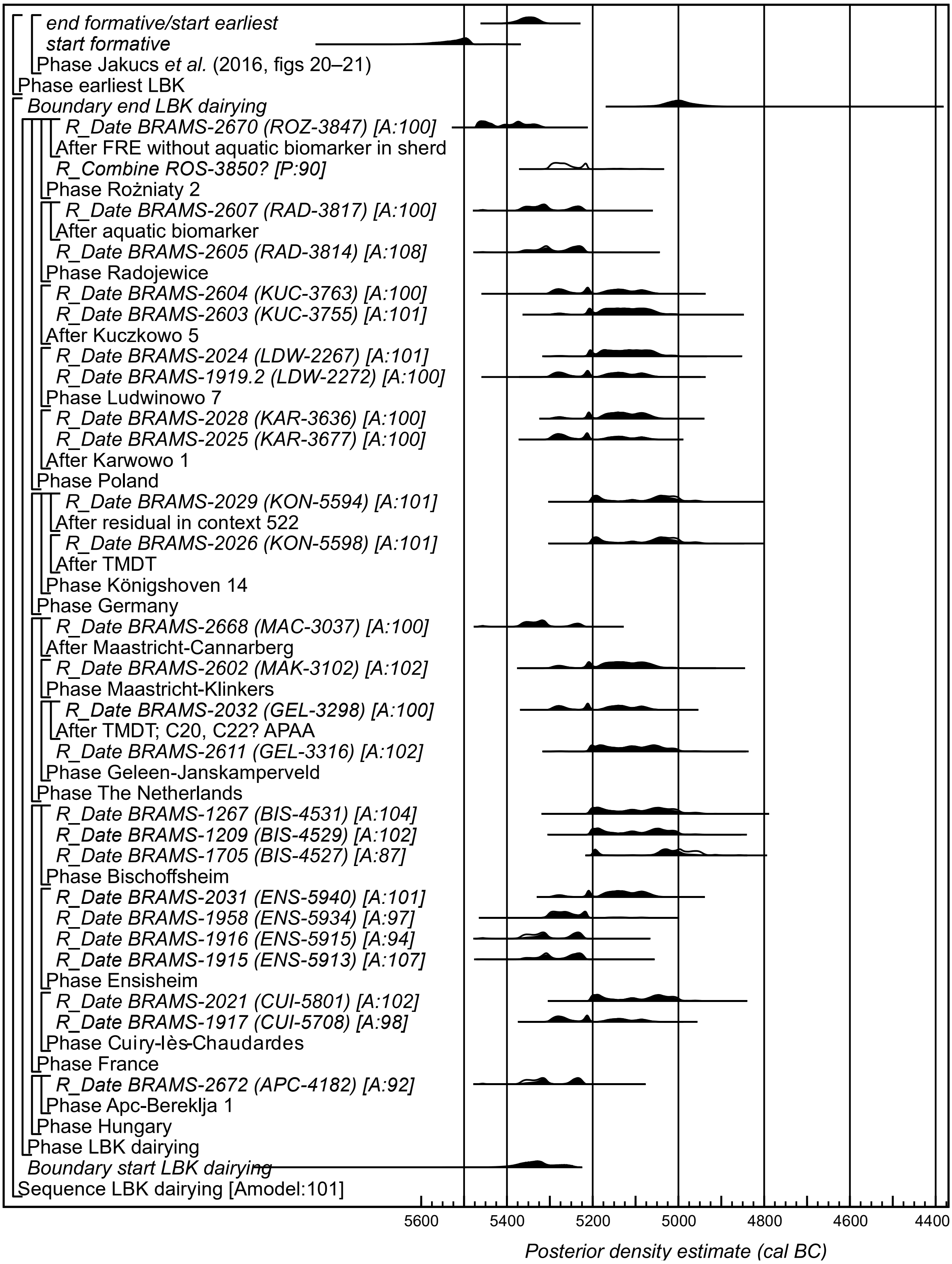

Figure C: Probability distributions of CSRA dates from lipid extracts from LBK pottery in the western and eastern areas shown on Figure 1. The format is identical to that of Figure 3. The large square brackets down the left-hand side of the diagram, along with the OxCal keywords, define the overall model exactly.

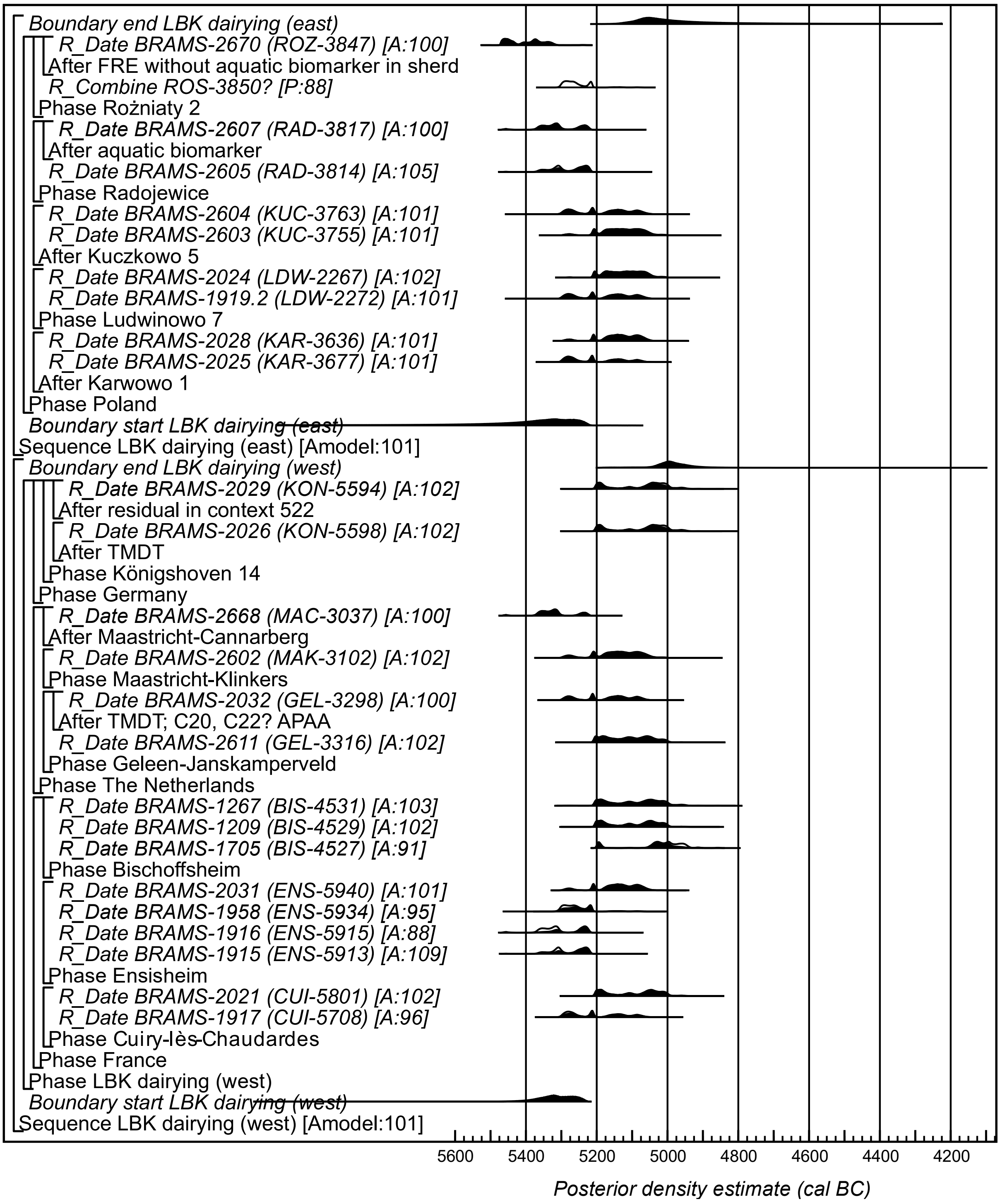

Supplement: Supplementary File [file pnas.2109325118.sapp.pdf]
